# GLULA: Linear attention-based model for efficient human activity recognition from wearable sensors

**DOI:** 10.1017/wtc.2024.5

**Published:** 2024-04-05

**Authors:** Aldiyar Bolatov, Aigerim Yessenbayeva, Adnan Yazici

**Affiliations:** Department of Computer Science, Nazarbayev University, Astana, Kazakhstan

**Keywords:** deep learning, human activity recognition, human–robot interaction, linear self-attention

## Abstract

Body-worn sensor data is used in monitoring patient activity during rehabilitation and also can be extended to controlling rehabilitation devices based on the activity of the person. The primary focus of research has been on effectively capturing the spatiotemporal dependencies in the data collected by these sensors and efficiently classifying human activities. With the increasing complexity and size of models, there is a growing emphasis on optimizing their efficiency in terms of memory usage and inference time for real-time usage and mobile computers. While hybrid models combining convolutional and recurrent neural networks have shown strong performance compared to traditional approaches, self-attention-based networks have demonstrated even superior results. However, instead of relying on the same transformer architecture, there is an opportunity to develop a novel framework that incorporates recent advancements to enhance speed and memory efficiency, specifically tailored for human activity recognition (HAR) tasks. In line with this approach, we present GLULA, a unique architecture for HAR. GLULA combines gated convolutional networks, branched convolutions, and linear self-attention to achieve efficient and powerful solutions. To enhance the performance of our proposed architecture, we employed manifold mixup as an augmentation variant which proved beneficial in limited data settings. Extensive experiments were conducted on five benchmark datasets: PAMAP2, SKODA, OPPORTUNITY, DAPHNET, and USC-HAD. Our findings demonstrate that GLULA outperforms recent models in the literature on the latter four datasets but also exhibits the lowest parameter count and close to the fastest inference time among state-of-the-art models.

## 1. Introduction

Human activity recognition (HAR) systems benefit from the valuable information provided by multimodal sensors, including acceleration, gyroscope, and temperature data. The challenge lies in identifying accurate and efficient classification methods for HAR systems, which find applications in diverse areas such as fitness monitoring and drug control systems (Khan et al., 2012), as well as stress and affect detection (Schmidt et al., 2018).

In HAR systems, the sensor signal time series are divided into equal-length subsequences using the sliding window technique. These subsequences are then classified into activities using various algorithms, ranging from traditional machine learning approaches like Support Vector Machines and Random Forest to advanced neural networks such as Recurrent Neural Networks (RNN), Convolutional Neural Networks (CNN), or hybrid models. Notably, deep neural network models have demonstrated superior performance in activity classification compared to conventional machine learning algorithms in HAR (Ma et al., 2019).

In addition to achieving high accuracy in activity classification, efficient resource utilization is essential in the field of HAR due to increasing computational demands. Therefore, the focus of our research is not only to improve the recognition of human activities but also to enhance resource usage by introducing a novel solution. Our proposed model is an attention-based non-recurrent architecture that incorporates gated convolutional networks (GCN) (Dauphin et al., 2017), a branching convolution structure, and linear attention. The GCN utilizes Gated Linear Units (GLU) as gate mechanisms, enabling convolutions without recurrent connections. To address constraints related to memory and model size, we introduce a learnable class token that is added at the beginning of the encoded data and employed in the classification layer (Tenney et al., 2019). This token, which has the same size as the embedding dimension, results in minimal parameter increase while maintaining effectiveness.

By integrating both local and global features, our proposed network eliminates the need for feed-forward (FF) layers, resulting in a substantial reduction in the number of parameters. This optimization takes into consideration the efficiency and complexity of inference. Furthermore, we introduce innovative features, such as the use of linearized attention instead of the conventional softmax self-attention, to further enhance the model’s performance.

Effective training of our network requires a significant number of samples, similar to other transformer-like structures. Instead of modifying the dataset or network architecture, we make adjustments in the training process. Specifically, we incorporate the manifold mixup regularization technique, which enables the learning of uncertainty and acts as a form of data augmentation (Verma et al., 2019). The generation of new samples using this technique enhances the efficiency of network training.

As discussed below, the attention-based linear model proposed for HAR tasks demonstrates improved performance while maintaining advantages in terms of size, space, and time complexities. In our study, we refer to this version of the network as GLULA. It outperforms various variants and state-of-the-art models across four benchmark datasets, while significantly reducing the number of parameters compared to recent state-of-the-art (SOTA) networks. The contributions of this article are as follows:

- We introduce a novel attention-based network architecture for HAR that offers several key contributions. Firstly, our model is parallelizable and achieves the lowest parameter count among novel works, optimizing space and time complexity through the utilization of linear attention. We also showed that the proposed method potentially has the fastest or close to the fastest inference time with respect to the input length among the latest models in the literature. This architecture demonstrates superior performance on four HAR datasets (SKODA, OPPORTUNITY, DAPHNET, USC-HAD) compared to recent models in the literature, and comparable results on the PAMAP2 dataset, validating the effectiveness of our proposed solution. We showcase performance improvements over recent state-of-the-art models using both benchmark test sets and the leave-one-subject-out (LOSO) cross-validation approach.
- Furthermore, we investigate the impact of different training techniques in scenarios where data is limited, which is often the case for HAR datasets. Our findings demonstrate that incorporating manifold mixups in a data-deficient environment enhances the network’s generalizability and achieves high-performance scores when combined with additional training procedures.
- Additionally, we conduct a comprehensive comparison of various layers at different positions within our proposed network. We illustrate that our network structure outperforms the different variants presented in this article. When comparing the softmax self-attention unit (Vaswani et al., 2017) with linear attention and gated convolutional networks, we observe that while softmax self-attention may have higher complexity, replacing linear attention or GCN with softmax self-attention in different parts of the network either yields equivalent or inferior performance. Furthermore, the utilization of linear attention improves the model’s speed compared to regular self-attention.
- Lastly, our architectural choices, such as discarding feed-forward networks and prepending learnable class tokens, result in minimal size overhead for the task. Our model showcases the lowest number of parameters (with a noticeable difference) compared to other state-of-the-art solutions while maintaining strong performance.

The remainder of the article is structured as follows. In Section 2, we provide an overview of related works in the field. The problem formulation and our proposed approach are outlined in Section 3. Details regarding the training and evaluation of our approach are presented in Section 4. In Section 5, we showcase the experimental results and conduct comparisons with other methods. Finally, in Section 6, we draw conclusions based on our findings and discuss potential avenues for future research.

## 2. Related work

HAR has been extensively studied using classical machine learning algorithms as well as deep learning models. Classical algorithms, such as decision trees, kNN, and Boosted classifiers, have been explored, with studies showing that kNN performs well on the PAMAP2 dataset (Reiss and Stricker, 2012). However, these classical approaches heavily rely on handcrafted features and often have limited effectiveness. In contrast, deep learning models, with their ability for automatic feature selection, have shown superior performance in HAR tasks, especially when utilizing CNN and LSTM architectures (Ma et al., 2019).

RNNs, while effective in capturing temporal dependencies, struggle with capturing long-range relationships due to the vanishing gradient problem. On the other hand, CNNs excel in parallelization but may face challenges in preserving spatial information. To address these limitations, researchers have proposed combined RNN-CNN models. However, such models often treat sensor modalities equally without considering their differences and importance. In response, Ma et al. (2019) introduced an attention mechanism to the CNN-RNN model, assigning weights to each modality and improving performance.

The Transformer architecture, initially introduced by Vaswani et al. (2017), has been successfully applied to HAR tasks. Transformers leverage non-recurrent self-attention, enabling parallelization and leading to improved performance in HAR. Building upon this concept, Mahmud et al. (2020) utilized a self-attention-based neural network for HAR, further enhancing performance. Additionally, the Evolved Transformer (EV) model proposed by So et al. integrates local information and expands data, which proves beneficial for capturing global features in HAR tasks (So et al., 2019).

In the context of HAR classification tasks, linear attention has emerged as a more efficient alternative to softmax self-attention models. Linear attention addresses issues related to complexity, performance, and memory requirements (Katharopoulos et al., 2020). Moya Rueda et al. (2018) proposed a deep learning model with parallel branches and temporal convolution, achieving high accuracy in HAR. Ma et al. (2019) introduced the multimodal AttnSense model, combining convolutional layers, attention mechanism, and GRU to capture spatiotemporal dependencies. Mahmud et al. (2020) addressed the challenge of parallelization by proposing a non-recurrent self-attention model. Furthermore, Tang et al. (2020) presented a memory-efficient CNN model with redesigned filters, reducing parameters while maintaining performance.

## 3. Methodology

### 3.1. Proposed approach

To provide context for the proposed architecture, it is essential to outline the problem at hand. Most HAR datasets exhibit a consistent structure, characterized by 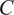 columns representing different sensor outputs. For instance, three columns may represent 3D acceleration data captured by an inertial measurement unit (IMU). Moreover, the IMU often includes additional sensors such as a magnetometer and an gyroscope. In HAR datasets, these sensor outputs are organized into 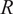 rows, with each row representing an instance of sensor readings over time. Consequently, we obtain a matrix 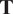 with dimensions 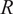 × 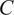 , encapsulating the time-series data from the sensors.

Then, 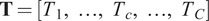 , where 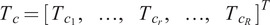 .

In this scenario, the atomic value of the sensor output at position (column) 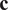 and time instance (row) 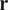 is denoted as 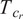 . The goal of the HAR task is to classify a label based on the given matrix 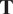 . The label can represent either a specific action or a sequence of actions.

The objective of this study is to develop an efficient non-recurrent model for HAR that achieves effective parallelization, considers the importance of each sensor output, and ensures high performance. Additionally, we aim to address the complexities associated with memory usage and inference time.

While Transformer networks have shown superior performance in classification tasks, they suffer from high time and space complexity. The Evolved Transformer model, discovered through evolutionary search, addresses some of these issues by reducing parameters and avoiding feed-forward constructions. However, EV models were primarily designed for conversational Natural Language Processing or translation tasks and still rely on softmax self-attention, which leads to high space and time complexity during inference.

To create a more efficient and suitable network for HAR, we draw inspiration from EV models and introduce significant modifications. Our approach incorporates the concepts of local–global aggregation and addresses the problem of quadratic space and time complexity by utilizing linearized attention.

**Figure 1.**
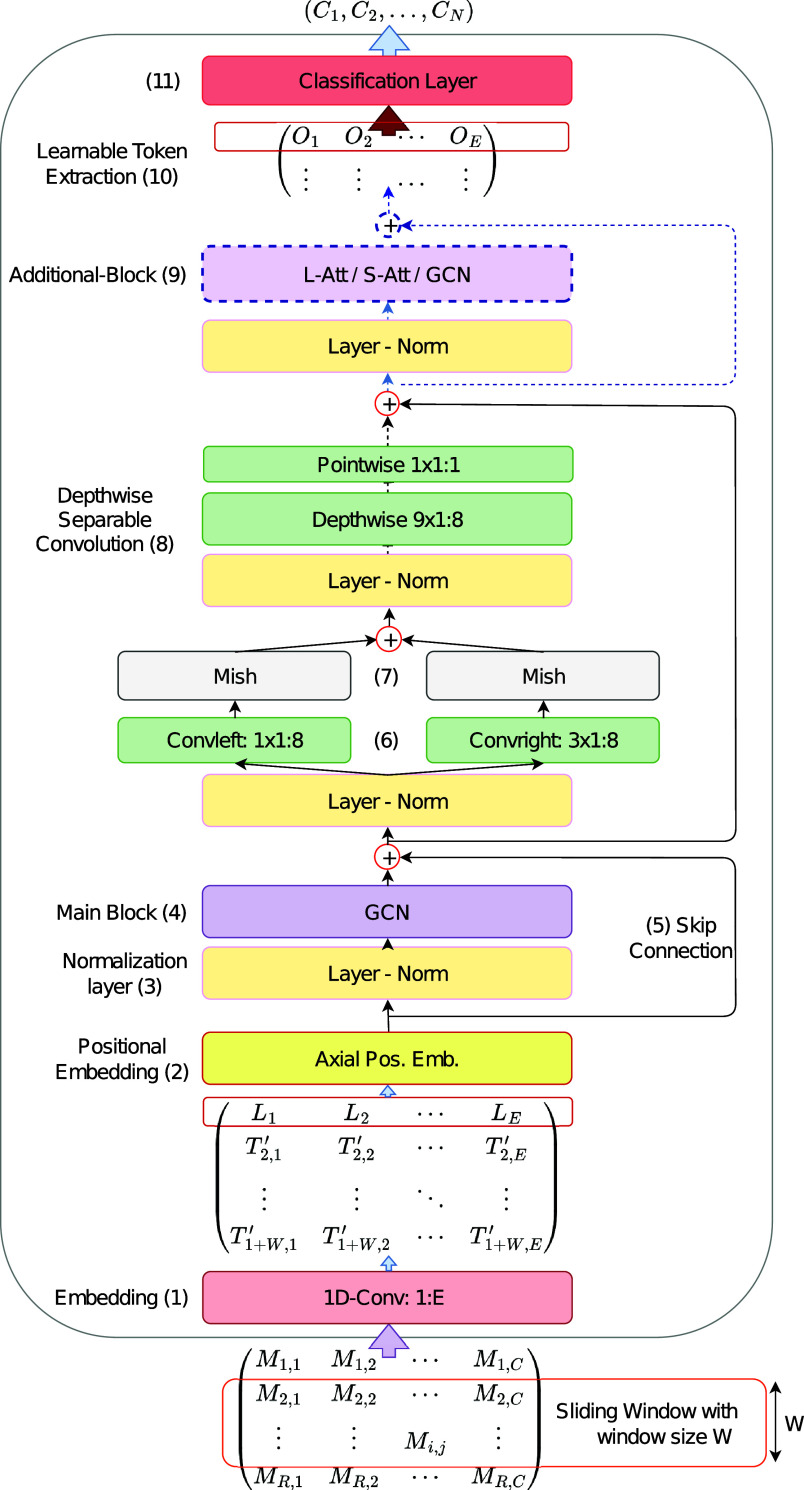
The graphical representation of the proposed model’s structure. Data preprocessing and each layer are shown and numbered following the model description given in the methodology. While the GCN, Linear Attention (L-Att), and Softmax Self-Attention (S-Att) can all potentially serve as the main block (4), GCN has been shown to outperform the others in this role, as highlighted in equation (1). Consequently, it is illustrated in the figure as the exclusive type for the main block. In contrast, for the additional block (9), all three network types (GCN, L-Att, S-Att) underwent full testing. Hence, the additional block in the graph showed as a choice among these three types. A comprehensive structure of each of the network types that was tested as (9) is provided in Figure 2.

Our novel HAR model begins by prepending learnable tokens (denoted as 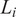 in Figure 1) and employs a gated convolutional network with a branching structure of wide convolutional layers. It also incorporates a linear attention network, which has linear complexity, to extract features for the classification layer. The classification layer consists of two fully connected layers that utilize the processed learnable class tokens, sized to one timestep.

However, like most transformer-based models, our approach requires a substantial amount of training data. Since many HAR datasets are limited in size, we employ manifold mixup regularization and other training techniques to enhance the network’s accuracy and generalizability in data-deficient settings.

The overall structure of our network is illustrated in Figure 1, with each component labeled accordingly. In the model, the input data is first segmented using a sliding window and then normalized. The normalization technique varies for each dataset. Next, each timestep of the normalized input data is mapped to a constant dimensionality 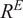 through a trainable projection or embedding, (1) in Figure 1. We append a learnable class token ( 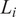 ) to the start of the input data, allowing it to extract relevant information from all timesteps and channels through the self-attention block. We demonstrate that a linearized version of softmax self-attention achieves effective results and learns to abstract and attend to the information, making the prepended learnable vector a valuable feature for classification.

To incorporate positional information and enhance the model’s performance on time-dependent tasks, we utilize axial positional embedding (Ho et al., 2019), which is learnable and added through augmentation, (2) in Figure 1. This approach factorizes the encoding matrix into two matrices, reducing the number of parameters and optimizing memory usage.

In the model, normalization layers are consistently applied prior to each block for distribution stability (Ba et al., 2016), exemplified by (3) in Figure 1. For the role of the main block, denoted by (4) in Figure 1, we evaluated various network types including GCN, Linear, and Softmax attention networks. In our experiments, the gated convolutional network demonstrates better generalization performance compared to softmax self-attention and linear attention. Additionally, skip connections, (5) in Figure 1, are used to facilitate gradient flow, partially addressing the vanishing gradient problem and promoting faster convergence (Drozdzal et al., 2016).

Next, the data is passed through two separate branches of convolutions, as shown in (6) in Figure 1, inspired by similar structures found in the EV. Various activation functions, such as ReLU and GELU, were evaluated, but the Mish activation function demonstrated the best performance in transformers, as depicted in (7) in Figure 1. Following the branching structure of EV models, a depthwise separable convolution is applied to extract spatiotemporal local features, as indicated by (8) in Figure 1.

**Figure 2.**
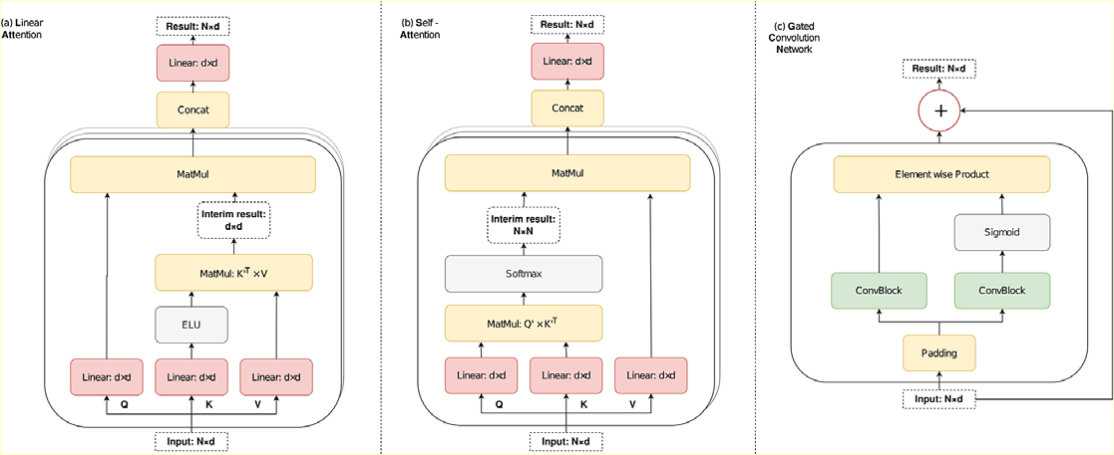
The graphical portrayal of each network type that was experimented as the additional block: Linear Attention (a), Softmax Self-Attention (b), and Gated Convolutional Network (c).

After this module, a skip connection is performed, and the data is then passed through an additional block, denoted as (9) in Figure 1, which significantly enhances the results by providing a more complex representation of temporal data. This critical block can be realized using one of the attention networks or GCN to produce the concluding data. In our study, the linear attention network outperformed GCN and softmax self-attention in the additional block. The linear attention network offers linear complexity with respect to the input length, while the complexity of softmax self-attention is quadratic. A comprehensive layout of each network variant (GCN, Linear-Attention, Self-Attention) that we experimented with for the additional block is illustrated in Figure 2.

Moreover, both softmax self-attention and linear attention networks have the same number of parameters, with GCN utilizing slightly fewer. However, during inference or training, the softmax self-attention network incurs a higher memory footprint and computational usage compared to the linear attention network.

Our assumption is that the gated convolutional network, serving as the main block, and the branched convolutions at the beginning of the model help capture local features and introduce new locally found information to the data. Subsequently, the linear attention network, as an additional block, extracts global features from the locally transformed data. Self-attention considers all data simultaneously, which may explain the superior performance of GCN in the main block and the utilization of attention in the additional block.

Finally, a learnable token is extracted and processed, as depicted in (10) in Figure 1, at the beginning of the processed input matrix. This token is then fed into the classification layer, shown as (11) in Figure 1. The classification layer includes the Mish activation function and, considering our concern for memory usage, the learnable token is of size one timestep or embedding dimension. The two fully connected layers of the classification layer scale accordingly to the token’s size, resulting in minimal additional parameter overhead.

Throughout the study, several models were tested, with a focus on three main variants: GLU-HAR, GLUSA-HAR, and GLULA-HAR. The “GLU” component represents the gated linear unit (or GCN), “SA” refers to self-attention, and “LA” denotes linear attention. The GLU-HAR model incorporates GCN as the additional block, while GLUSA-HAR utilizes softmax self-attention, and GLULA-HAR employs the linear attention network. As previously mentioned, based on the empirical evaluation, all three use GCN as their main block. In some tables, the HAR appendage is omitted for brevity.

In summary, our proposed architecture enhances the efficiency and performance of HAR models by taking into account the unique characteristics of sensor data. Through the utilization of linearized attention, axial positional embedding, and manifold mixup regularization, we address challenges related to space complexity, time complexity, positional information, and limited training data, thereby contributing to the advancement of HAR techniques. As discussed later, GLULA-HAR achieved the highest performance among the three variants, outperforming other models and demonstrating comparable or superior results compared to state-of-the-art approaches. Further details on these models and their outcomes can be found in Sections 4 and 5.

### 3.2. Gated convolutional network

In line with the aforementioned information, it is worth noting that RNN suffers from a lack of parallelization in input processing, resulting in slower training and inference times. On the other hand, CNN networks can perform computations simultaneously, making them faster than RNN-based solutions. To create more efficient language models, Dauphin et al. (2017) introduced a gated convolutional network that utilizes parallelizable causal convolutions.

Let us begin by clarifying what causal convolutions entail. Causal convolutions are similar to regular convolutions, but the input is left-padded with zeros by 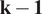 , where 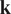 represents the kernel size of the causal convolution block. This approach ensures that the GCN only considers previous and current timesteps, avoiding any influence from future inputs.

The input 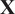 would be fed into two different causal convolutional blocks with filters 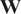 and 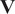 , respectively, where 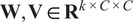 and *C* is the dimension size of sensors’ signal. Then, two separate outputs will be put into the gated block, which uses a mechanism of gating linear unit (Dauphin et al., 2017). This mechanism puts one of the outputs through the activation function and then gates the other by element-wise multiplication. See formula (1), where 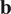 and 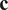 are learnable bias parameters; 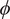 is an activation function and 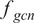 is a function interpretation of a simple GCN.
(1)

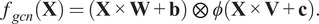

In concept, the gating mechanism can perform the selection of valuable features by 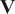 that control what information from other output (which was convolved by 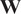 ) will be passed to the subsequent layers. By this, GCN learns to move only relevant information and gain non-linearity. Furthermore, as reported by the original paper (Dauphin et al., 2017), the residual skip was added to the GCN for reducing the vanishing gradient problem, and GCN was with bottleneck structure within a layer for reduction of computational cost. For the activation function, 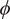 , the Mish function was used as it showed optimal results in a variety of tasks (Misra, 2019).

### 3.3. Self-attention

The self-attention mechanism can be viewed as a means of computing the significance or importance of each timestep in a sequence. In our scenario, the self-attention function establishes relationships between each timestep and all other timesteps within the input. By assessing the similarities and correlations between timesteps, self-attention calculates new values for each timestep. These values reconstruct the timesteps and incorporate information from other timesteps based on their relevance.

As detailed by Vaswani et al. (2017), the query, key, and value (*Q*, *K*, *V*) can be formed by linearly transforming the source sequence using three distinct learned weight matrices 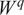 , 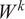 , and 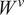 . The query can be envisioned as the information being sought, while the key represents the data that is pertinent to this information. The value, on the other hand, is a learned representation of the content within the input.

In our case, we represent the query and the key as separate transformed timesteps, which are subsequently compared. This comparison is accomplished through a scaled dot-product, following the approach outlined in Vaswani et al.’s (2017) paper. The output of this comparison represents the similarities between the query and key, or in other words, the attention scores among different timesteps. Next, the output is normalized using softmax and multiplied by the value vector, resulting in values that encode the relative importance of each timestep and incorporate relevant information from other timesteps.
(2)

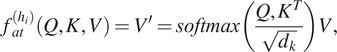

where
(3)

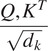

is the compatibility function in the form of a scaled dot-product, 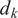 is the dimension of the key used for scaling the function to improve numerical stability, 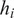 represents head *i.* After computing attention on *Q*, *K*, and *V*, we get an output matrix, which can then further be utilized as an attention head. We can compute more heads and by this, we can make use of multi-head attention. Distinct heads can capture different unique features by having individual parameters. Then, to get the final result, the output from different heads is concatenated, and by applying learned linear projection 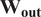 , the concatenated outputs are transformed into the original dimension.
(4)

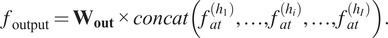



### 3.4. Linear attention

In the softmax self-attention mechanism, once the query, key, and value (*Q*, *K*, and *V*) are obtained through linear transformations, all three are input into equation (2). In our study, the dimensions of *Q*, *K*, and *V* are 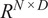 , where 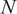 represents the sequence length of the input, and 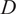 denotes its dimensionality. Examining equation (2), we can observe that softmax attention scales quadratically with respect to 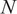 , resulting in a computational complexity of 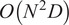 (Katharopoulos et al., 2020). This quadratic scaling is also applicable to memory consumption since the full attention matrix 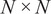 needs to be stored for gradient computation.

To alleviate the time and space complexity, it is essential to consider softmax self-attention as a more generalized form of self-attention, where the similarity function is an exponentiated dot product between the query and key (*Q*, *K*). By introducing a unique value vector *V′*, the generalized self-attention can be expressed as follows:
(5)

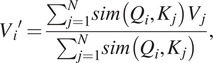

where, in the softmax self-attention case, the similarity function 
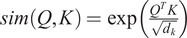
 as it was stated above. To note, in the self-attention different similarity function can be used such as polynomial attention (Tsai et al., 2019).

For equation (5) to be an attention function, a constraint must be followed for a 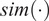 function: to be a non-negative function (Katharopoulos et al., 2020). This actually includes all kernels of type 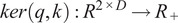 . Then given such a kernel 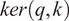 with a feature mapping 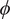 , we can define 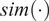 the function as the corresponding kernel 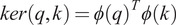 . Then we can rewrite the whole equation (5) as follows,
(6)

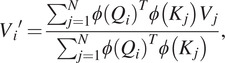

then, using the associative property of the matrix product, the equation goes further:
(7)

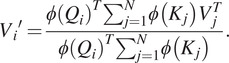


Equation (7) is called linear attention. It has linear complexity in time and memory with respect to the sequence length 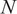 , due to 
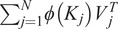
 and 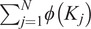 can be computed once for each query (Katharopoulos et al., 2020).

For linear attention, feature maps of a certain dimensionality 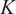 are first computed, which in turn give us the complexity 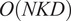 for linear attention in terms of mathematical operations. However, Katharopoulos used an exponential linear unit as the feature map (Katharopoulos et al., 2020). The full equation looks like this: 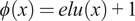 . In linear attention, this feature map resulted in a complexity of 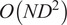 in terms of mathematical operations. It is very efficient if 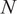 is considerably greater than 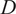 . As the authors have shown in their work, linear attention achieved results comparable to regular softmax self-attention while being faster on the task where the length of the sequence is higher than the dimensionality of the data (Katharopoulos et al., 2020).

### 3.5. Training techniques

The development of Manifold Mixup regularization was driven by the need to address the issue of overconfident predictions made by neural networks trained on hard labels (Verma et al., 2019). Overconfidence can be problematic as it may result in incorrect classifications when evaluated on slightly different samples, which can include outliers, noise, or distribution shifts.

To mitigate these effects, manifold mixup regularizers aim to encourage deep learning models to produce less confident predictions during training by leveraging the interpolation of hidden features as an additional training signal.

Algorithm 1 Manifold mixup regularizer (Verma et al., 2019)

**Input:** Deep neural network 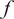 with set of layers 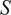 and parameters 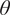 , constant 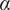 , input minibatches.

1. Random layer 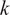 from 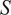 is selected.
2. Minibatches 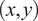 and 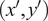 is fed through the layers of the network 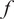 until layer 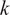 . The resulting hidden representations are 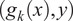 and 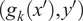 . Minibatches could be two distinct or the same reshuffled batch.
3. Input Mixup 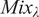 is performed on intermediate hidden representations 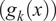 , 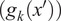 and one-hot labels ( 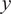 , 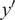 ). The result is the mixed minibatch: 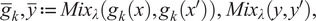 where 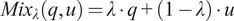 and 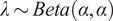 .
4. The forward pass is continued in the model from where we stopped at the layer 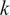 until the output using newly acquired mixed minibatch.
5. The output and mixed labels 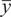 are fed into the loss function, and the calculated value is used to update all of the parameters 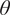 in 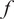 . During updating, backpropagation goes through the whole computational graph.

**Output:** Neural network 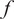 with updated 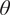 .

In order to represent this regularization technique, we first define a deep neural network as 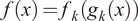 , where 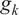 represents a component of the network that maps the input 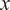 to the hidden representation at a specific layer 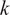 . The function 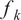 encompasses the remaining parts of the model that lead to the output 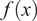 based on the extracted features 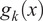 . To incorporate Manifold Mixup as a regularization approach for training such a network, we need to follow five steps.

By employing Manifold Mixup during training, the resulting network exhibits smoother decision boundaries across various levels of representation, as noted in Verma et al.’s (2019) work. Moreover, the network learns flattened class representations with reduced variance directions. These effects ultimately enhance the model’s generalization capabilities, leading to improved performance not only on test data but also in the face of adversarial attacks (Verma et al., 2019).

We also considered Manifold Mixup as a valuable data augmentation technique, particularly in scenarios where datasets are limited, such as in the case of HAR datasets. The rationale behind utilizing Manifold Mixup is that it generates new mixed samples at each step, which differ not only due to changes in mini-batches but also as a result of shuffling and mixing at various layers. Hence, we applied and evaluated this regularization method in our work.

Another challenge associated with limited datasets is the issue of overfitting and divergence. Overfitting arises from training on a small sample space, but we partially addressed this problem by incorporating Manifold Mixup. However, to further mitigate divergence between re-initialized networks, scheduling techniques can be employed. These techniques promote more stable training and weight updates. In our study, we utilized the one-cycle policy proposed by Smith (2018). This policy involves gradually increasing the learning rate to a maximum value and then annealing it close to zero. The result is that it helps the model navigate steep points of the loss landscape and settle into flatter minima, enhancing stability during training.

## 4. Experiment evaluation

The section begins by presenting the setup and performance measurements. Subsequently, we provide details regarding the datasets and preprocessing techniques employed. The final subsection outlines the training procedures implemented and the hyperparameters utilized in the experiments.

### 4.1. Setup and evaluation

Our proposed solution was implemented using the PyTorch library and trained and tested on a cloud-based GPU. The network was randomly initialized, and a batch size of 64 was utilized. To ensure robustness, each experiment was repeated five times with different seeds, and the averaged values from these experiments were used in the tables for analysis.

For evaluating and comparing the models’ performance, we employed the weighted F1-score as the measurement metric. The weighted F1-score takes into account the label imbalance of HAR datasets and is independent of the class distribution (Tang et al., 2020). Similar to the macro F1-score, the weighted F1-score assigns a weight to each class, which corresponds to the class’s sample proportion in the total dataset:
(8)

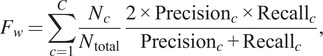

where
(9)

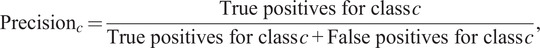



(10)

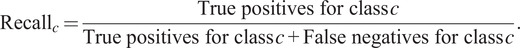



### 4.2. Datasets

To evaluate the proposed model, its variations, and different training techniques, we utilized five HAR datasets for our experiments.

The first dataset used for benchmarking is PAMAP2 (Reiss and Stricker, 2012). PAMAP2 consists of sensor data from three IMUs placed on the chest, the dominant leg’s ankle, and the wrist of the dominant arm. Each IMU includes an accelerometer, gyroscope, magnetometer, and temperature sensor. Heart rate measurements were also recorded separately. The sensors had a sampling frequency of 100 Hz, except for the heart rate which was sampled at 9 Hz. PAMAP2 was collected from 9 participants and contains 12 different activities, with an additional 6 activities that were not used. Activities include basic postures, locomotions, household chores, and recreational/sports. Each type of activity was collected in different sessions with a time break between them. Following the principle of leaving-one-subject-out as done in previous works (Tonmoy et al., 2021), we used data from participant number 106 as the benchmark test set, while the rest of the dataset was used for training.

The second dataset is SKODA (Stiefmeier et al., 2008), which focuses on describing the activities of workers in a car manufacturing environment. The dataset includes several accelerometers worn by a worker, with a sampling frequency of 98 Hz. Data was recorded from a single subject for all cases. The dataset comprises 10 different activities performed during the auto manufacturing process, along with a null division representing no activity. In total, there are 11 classes. These activities range from manual documentation tasks (like writing on a notepad) to specific inspection or manipulative actions related to different parts of a vehicle. For training, 90% of each class was used, while the remaining 10% was reserved for testing.

The third dataset is OPPORTUNITY (Roggen et al., 2010), which contains data from body-worn and ambient sensors, with each timestep annotated with a specific activity. Activities are annotated at three levels: high-level, mid-level, or gestures, and low-level or modes of locomotion. For our experiment, we focused only on the mid-level activities, while other activities were labeled as null. This resulted in a total of 18 different activities, with a significant class imbalance where around 75% of the dataset consists of the null class. The dataset includes one drill session and five daily activity (ADL) sessions performed by the subjects. Drill sessions involve the subject carrying out a specific sequence of actions, such as opening and closing various kitchen doors and engaging in cleaning and drinking from various positions. In ADL routines, the individual performs broader tasks like waking up, grooming, preparing breakfast, and cleaning, but has more flexibility in the order of these specific actions. The sensors were sampled at a frequency of 30 Hz. Following previous research, we used the fourth and the fifth ADL sessions performed by subjects 2 and 3 for testing, while the rest of the dataset was used for training and validation.

The fourth dataset is USC-HAD (Zhang and Sawchuk, 2012), which provides sensor data from body-worn gyroscopes and accelerometers. Each sensor provides 3-axis readings, resulting in a total of six dimensions for each instance of data. The sampling rate is set at 100 Hz. The dataset consists of an equal number of male and female participants, with each subject performing 12 different activities. These activities span from dynamic motion tasks like walking, jumping, and running to various stationary postures. USC-HAD is a challenging dataset due to the diversity of activities, sensor placement, and low dimensionality, which limits the available activity information. However, the dataset size is larger compared to the other three datasets, and it is also balanced unlike OPPORTUNITY, which is heavily skewed toward the null class. Following the LOSO principle, two subjects (13 and 14) were separated for testing.

The last dataset is DAPHNET (Bachlin et al., 2009), which was collected to evaluate the ability of different machine learning methods to learn and recognize gait freeze events. This dataset has the potential for developing an assistant for Parkinson’s disease patients. The data includes readings from three wearable acceleration sensors placed on the hips and legs, resulting in a total of nine channels per sample. Each sample is annotated as either a freeze or not. DAPHNET shares similar challenges with USC-HAD in terms of sensor placement and low dimensionality, which limits the available information for activity prediction. However, the dataset is imbalanced toward the no-freeze class and has only two classes to recognize. The sensors were sampled at a frequency of 64 Hz. Following previous works, we used subject 2 as the benchmark test set (Tonmoy et al., 2021).

A summary of each dataset, including key information and an outline, can be found in Table 1.

**Table 1.** Information about presented datasets’ structure

Dataset	Number of activities	Number of subject(s)	Test subject(s) ID
PAMAP2	12	9	106
SKODA	11	1	10% of each class
OPPORT.	18	4	2,3(Run 4,5)
USC–HAD	12	14	13, 14
DAPHNET	2	10	2

### 4.3. Window sizes’ analysis

Window sizes can differ, but changing them alters the dataset’s sample count and activity distribution. As a result, comparing models that use different window sizes for evaluation is not equitable.

We have also considered performance relative to different window sizes, informed by existing research. For instance, in Ma et al. (2019), the authors analyzed the impact of window size on final scores. Their findings showed that a smaller sliding window typically leads to weaker recognition accuracy. Optimal performance was achieved with 15 and 20-width sliding windows for Skoda and PAMAP2, respectively. In Mahmud et al. (2020), the model they introduced was only slightly affected by window size changes. However, they did notice that datasets with complex activities needed a more extended sliding window for capturing the correct activity label.

Thus, by taking into account the prior analyses and ensuring a fair comparison, we have selected window sizes for each dataset based on those used in benchmark models and recent works in the literature.

As for computational performance, the window size has a direct linear effect on inference speed due to our solution’s design. As outlined in the manuscript, many of the GLULA layers possess a linear time complexity concerning input length. That includes the additional block (Linear Attention) and main block (GCN). So, if the window size is doubled, the input length also doubles, roughly doubling the inference time. However, there is always some computational overhead, so it will not exactly double the time, but close to that. This can be prominently seen in Table 4. Nevertheless, the model size (the number of parameters) remains constant independently of the window size. The reason is that the number of parameters depends on the embedding dimension, which, in turn, is related to the sensor output dimensionality, not the window size.

### 4.4. Data preprocessing

All the presented datasets contain NaN values, which occurred during the recording process. These NaN values indicate instances where sensors were not functioning properly due to various reasons such as loss of connection, internal errors, or inability to maintain the sampling frequency.

NaN values can have a significant impact on the computations of the model and can potentially corrupt the results. Therefore, as a preprocessing step, the data were linearly interpolated to replace the NaN values with estimated values based on neighboring samples.

Furthermore, it was observed empirically that normalized data tends to exhibit more stable behavior during training and can accelerate the convergence of the model. To achieve this, all the datasets were subjected to *Z*-score normalization, which standardizes the data distribution.

Next, the data were segmented using a sliding window technique with a 50% overlap. The window size for PAMAP2, DAPHNET, and OPPORTUNITY was set to the standard value of 5 s in real-time, as used in previous works (Tonmoy et al., 2021). For the SKODA dataset, the window size was set to 2.5 s. In the case of USC-HAD, the window size was set to 1 s, which is the standard time span utilized in related studies (Haresamudram et al., 2020; Mahmud et al., 2020). After segmentation, each resulting sequence was fed into the network for classification, where the model performed predictions on each segment.

### 4.5. Hyperparameters and training

Firstly, various attention networks were tested as the main block in the model, but they did not yield any improvement in performance. Moreover, they consumed more memory and time compared to GCNs.

To evaluate the performance of different network configurations in the additional block layer, three types of models were constructed as described in Section 3. The first model, called GLU-HAR, utilized GCNs as both the main block and the additional layer. The second model, GLUSA-HAR, used a GCN as the main block and a self-attention block as the additional layer. The GLULA-HAR model shared the same structure as GLUSA-HAR but employed a linear attention network as the additional layer.

Two other models, namely GLUDynamic-HAR and GLUlightweight-HAR, were not included in the resulting tables and were not evaluated with other parameters. These models followed a similar structure to GLUSA-HAR, but differed in the additional layer, utilizing dynamic layers and lightweight convolutional networks, respectively (Wu et al., 2019). However, the results obtained from these models were significantly inferior to the scores achieved by GLUSA-HAR, and thus they were not further tested.

In the model, the input data is embedded into a new dimensionality from the original number of channels. After resizing, it is important to ensure that the attention layer utilizes different heads to capture distinct features. To achieve this, the embedding dimension 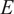 was set to be a power of two. Specifically, we computed 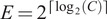 , where 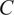 represents the number of channels. The count of model parameters differs proportionally to 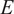 for each dataset. In the case of USC-HAD, where the channel set is limited, the model size is smaller. To enhance its learning capabilities, the embedding dimension was doubled. Despite this modification, the number of parameters for USC-HAD remained comparatively low. More details can be found in Section 5.2.

A comparison between the Adam and AdaBelief optimizers was conducted in Section 5.1. Both optimizers performed well, but AdaBelief showed better performance. AdaBelief is a combination of Adam and stochastic gradient descent (SGD), providing flexibility to adapt to different problem scenarios. Additionally, AdaBelief takes into account the gradient and the curvature of the loss function. Based on the test results and the aforementioned reasons, all experiments utilized the AdaBelief optimizer during training.

As mentioned earlier, HAR datasets can be imbalanced, as in the case of OPPORTUNITY, and have a limited sample size, which is crucial for self-attention-based models. However, the opposite holds true for USC-HAD, where there is an equal number of examples per class and a large number of samples. Therefore, Manifold Mixup and input balancing techniques were employed for PAMAP2, SKODA, DAPHNET, and OPPORTUNITY.

In GLUSA-HAR and GLULA-HAR, two attention heads were utilized for PAMAP2, SKODA, and OPPORTUNITY. However, for USC-HAD and DAPHNET, which have a small number of channels and consequently a small embedding size, only one attention head was used. Nevertheless, using two attention heads for these datasets would not noticeably degrade performance. Increasing the embedding size to make the model wider in all dataset cases, as mentioned before, did not lead to any significant changes.

## 5. Results of the experiments

Initially, in 5.1, training techniques were assessed on our proposed models, GLULA and GLUSA. Subsequently, three variations of the model were trained using the suggested training methods and compared across HAR datasets in Section 5.2. Additionally, the impact of the network structure on inference time was examined.

In Section 5.3, we provide a brief description of the networks employed in other HAR papers. These networks are then compared with our proposed model in Section 5.4, utilizing benchmark test sets. Furthermore, in Section 5.5, a comparison is conducted using the leave-one-subject-out cross-validation technique. In Section 5.5, we also delve into an experiment focused on performance speed comparisons. To emphasize the efficiency and acceleration achieved by our proposed method in contrast to contemporary state-of-the-art models, experiments were conducted comparing inference times. These comparisons utilized the official implementations of the networks outlined in Section 5.3.

### 5.1. Evaluation of training methods

Before evaluating the proposed model and its variations with the suggested training techniques, it is important to test the performance of each training method individually. For this purpose, the PAMAP2 dataset was selected as it provides a good balance among the different datasets. PAMAP2 is moderately imbalanced, but not as severely as OPPORTUNITY, and it has a sufficient sample size for training, although smaller than USC-HAD. This makes PAMAP2 a suitable benchmark for comparing the performance of our model under different training conditions and assessing how well the suggested methods meet the proposed expectations. Both GLULA and GLUSA models were used for testing the training techniques, while GLU-HAR was not considered due to its overall poor performance, as shown in Section 5.2.

As depicted in Table 2, manifold mixup (MM) significantly improved the performance of GLUSA. With GLULA, although the F1-weighted score remained nearly the same, the macro-score increased from 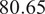 to 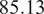 . This indicates that MM helped the model better capture class differences, while the weighted score showed minimal change. It is worth noting that mixups can be seen not only as a regularization technique but also as an augmentation method. Such an approach can be particularly beneficial in scenarios with limited data or imbalanced class distributions. Therefore, it can be effectively employed to address the specific requirements of HAR tasks, as demonstrated in our results.

**Table 2.** F1-weighted scores with STD on PAMAP2 using training methods on GLULA and GLUSA models

Methods	Models	F1w	STD
Adam	GLULA–HAR	86.69	3.18
	GLUSA–HAR	87.15	3.91
Adam + MM	GLULA–HAR	86.04	3.48
	GLUSA–HAR	88.54	2.52
Adam + MM + Scheduling	GLULA–HAR	88.08	2.89
	GLUSA–HAR	88.12	2.38
AdaBelief + MM + Scheduling	GLULA–HAR	90.09	1.14
	GLUSA–HAR	89.89	2.16

Furthermore, as observed in Table 2, scheduling techniques contributed to more stable training and reduced divergence across multiple observations (five in our case). This is evident from the lower standard deviation (STD) observed across the results of the experiments. With more stable training, the average score of GLULA improved to 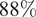 , as it no longer had a lower offset than before.

When evaluating the results of using the AdaBelief optimizer, it is evident that the accuracy scores for both GLULA and GLUSA models improved significantly. The training process of the models benefited from AdaBelief’s capability to consider both the curvature of the loss function and its gradient. Additionally, another advantage of incorporating the AdaBelief optimizer is its tuning capacity, which allows for further optimization. In the other datasets, the inclusion of AdaBelief resulted in nearly identical results for both GLUSA and GLULA models.

### 5.2. Evaluation of proposed models

First, it can be observed from Table 3 that GLULA and GLUSA models have the same parameter count for each dataset. This similarity arises due to both linear attention and self-attention layers having an equal number of learning parameters. However, they differ in terms of time and space complexities during inference. Linear attention approximates the computations of softmax self-attention, resulting in linear complexity with respect to the input length but without reducing the parameter count. On the other hand, GLU-HAR has a slightly larger parameter count.

**Table 3.** Results obtained on different datasets using the proposed GLULA and its variations

Dataset	Models	F1w	Num. of Param. (K)
PAMAP2	GLULA–HAR	90.09	50.2
	GLUSA–HAR	89.89	50.2
	GLU–HAR	64.05	54.4
SKODA	GLULA–HAR	97.63	51.4
	GLUSA–HAR	97.76	51.4
	GLU–HAR	88.77	55.6
OPPORT.	GLULA–HAR	95.93	196
	GLUSA–HAR	95.50	196
	GLU–HAR	87.50	213
USC–HAD	GLULA–HAR	59.38	4.0
	GLUSA–HAR	54.26	4.0
	GLU–HAR	38.31	4.3
DAPHNET	GLULA–HAR	94.11	3.8
	GLUSA–HAR	92.38	3.8
	GLU–HAR	80.69	4.1

Overall, despite the similar structure, the networks’ sizes are nearly identical, but they exhibit significant differences in their complexities. GLULA-HAR is faster than GLUSA-HAR, as reflected in the speed comparison shown in Table 4. However, the most crucial factor is performance, as indicated by the 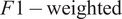 scores. As seen in Table 3, GLULA-HAR outperforms GLUSA-HAR by a small margin on the PAMAP2 dataset. Conversely, GLU-HAR performs substantially worse than the other two models, with a score of 64.05%.

**Table 4.** Speed comparison using the average forward pass time of our model with its variations on different datasets

Model	PAMAP2	SKODA	OPPORT.	USC-HAD	DAPH.
GLU	34.3	17.7	18.6	6.82	20.38
GLULA	35.2	18.1	18.8	6.84	20.43
GLUSA	42.8	19.5	19.4	7.53	22.1

*Note.* The unit is milliseconds (ms).

Table 3 demonstrates that GLUSA-HAR and GLULA-HAR have almost the same score on the SKODA dataset. The results exhibit a slight fluctuation of 0.13%, with GLUSA-HAR having a slight advantage. Once again, GLU-HAR achieves the lowest score. In both the PAMAP2 and SKODA datasets, the embedding size is the same due to the number of channels, resulting in approximately 50 K parameters. The only minor difference arises from the classification and embedding layers.

For the OPPORTUNITY dataset, the segments have the same number of timesteps as PAMAP2, but the number of sensor dimensions is tripled, and the embedding size is doubled. Consequently, the models are larger in size. Similar to SKODA, GLUSA-HAR, and GLULA-HAR show minor differences in performance, with GLULA-HAR leading by 0.43% with a score of 95.93.

The USC-HAD dataset is the most complex, with the lowest number of channels and a small-scale embedding dimension. Even after doubling the embedding dimension, the number remains small, around 4.0 K. In this case, GLU-HAR once again performs the worst. GLULA-HAR outperforms the softmax self-attention-based model by 5.12%, achieving a 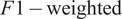 score of 59.38%. Attempts to improve the model’s performance on USC-HAD by adding more learnable tokens or increasing the embedding dimension resulted in a size increase but degraded network performance due to overfitting. Therefore, the number of parameters for USC-HAD is optimal, and the embedding cannot extract more valuable information even with a low count.

In the DAPHNET dataset, the embedding dimension is the same as in USC-HAD, which is 16. This leads to an almost identical number of parameters between the DAPHNET and USC-HAD cases, around 4.0 K. However, what makes the DAPHNET task easier compared to USC-HAD is that there are only two activity classes: gait freeze and not. Similar to USC-HAD, GLU-HAR performs the worst, while GLULA-HAR outperforms the self-attention-based model by a significant margin, with a score of 94.11% for the linear-attention-based model compared to 92.38% for GLUSA-HAR on the benchmark test set.

Although softmax self-attention is a powerful mechanism, it may lead to overfitting in constrained environments. In contrast, linear attention, despite being an approximation, often provides a more general solution. This is evident in the significantly higher weighted F1 scores achieved by GLULA-HAR in the USC-HAD and DAPHNET datasets, and comparable results to the GLUSA model on other benchmarks. Therefore, in the context of HAR, linear attention proves to be more effective.

Additionally, in theory, GLULA-HAR should be faster during both forward and backward propagation compared to GLUSA-HAR. To verify this, we examine the speed of the models, as presented in Table 4.

We conducted a comparison of the inference times for each network on different datasets, using a cloud-based system. It is important to note that the time values provided may vary across different machines. The measurements are given in milliseconds, with lower values indicating better performance.

As shown in Table 4, GLULA-HAR consistently outperforms GLUSA-HAR in terms of speed across all datasets. This advantage is more pronounced when the ratio between input dimensionality and input sequence length is lower. Notably, this effect is particularly evident in the PAMAP2, DAPHNET, and USC-HAD datasets, where the ratio is the lowest. Although GLU-HAR was the fastest among the models, its poor performance and higher number of parameters outweigh this advantage. Therefore, this variation of the model is considered the least favorable.

While the inference times of the models were relatively close in our cloud-based GPU setup if we were to reduce the allocated memory and bandwidth or increase the batch size, the speed difference between GLULA-HAR and GLUSA-HAR would become more pronounced. This indicates that in computationally limited environments, such as embedded systems, GLULA-HAR would be even faster than GLUSA-HAR.

Considering both the speed results and the performance scores from Table 3, we can conclude that the GLULA-HAR model is the preferred choice among the different variations of the proposed solution. Furthermore, it offers the advantage of linear complexity compared to the quadratic complexity of GLUSA-HAR.

### 5.3. Compared algorithms

We conducted a comparison between our proposed models, GLULA-HAR and GLUSA-HAR, and several other existing methods:

*Lego-CNN* (Tang et al., 2020): A lightweight convolutional neural network that employs memory-efficient lego-filters.

*Self-Att* (Mahmud et al., 2020): A self-attention-based model that utilizes SA blocks along with sensor Modality Attention and global Temporal Attention. The evaluation of this model differs from others as it uses the 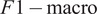 score instead of 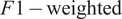 . We followed the structure of the model and estimated the number of parameters. However, we could not reproduce the results on the PAMAP2 dataset due to a minor code oversight, where the label class was included as input during inference instead of being predicted. Therefore, we relied on the results presented in the papers (Mahmud et al., 2020; Tonmoy et al., 2021).

*DeepConvLSTM* (Ordóñez and Roggen, 2016) (also known as DCL): A classical approach for HAR tasks that combines convolutional layers with recurrent units. The exact number of parameters may vary depending on different implementations. This model can be trained effectively with limited data, unlike self-attention-based models and transformers. Hence, we referred to the results presented in Ma et al. (2019) and Tonmoy et al. (2021).

*Attsense* (Ma et al., 2019): A multimodal model that incorporates attention-fusion subnets. It combines convolutional layers, Gated Recurrent Units, and attention mechanisms. This model extends the DeepConvLSTM and DeepSense models, and the number of parameters is similar to DeepConvLSTM. However, due to its reliance on recurrent mechanisms, it is less computationally efficient in terms of inference and training speeds.

*R-CNN* (Moya Rueda et al., 2018): A convolutional neural network that has demonstrated high performance on various datasets. Although the original work did not cover all the datasets we used, we employed the official implementation and extracted the number of parameters.

*HSA* (Tonmoy et al., 2021): This model addresses the challenge of recognizing unseen activities through a hierarchical self-attention-based approach. It combines data from different sensor placements over time and incorporates a decoder that uses feature representations from the self-attention encoder for detecting unseen activities in open-set recognition. However, the encoder-decoder structure of hierarchical self-attention adds complexity. Similar to the Self-Att model, there was a misstep in the PAMAP2 dataset implementation where labels were included as part of the input during inference. We relied on the results from the paper (Tonmoy et al., 2021), but this oversight should be addressed in future works.

*iSPLI* (Ronald et al., 2021): A deep learning model inspired by the Inception-ResNet structure introduced by Google. It emphasizes high predictive accuracy while utilizing fewer device resources. The authors tested the architecture on four datasets using transfer learning and believe that their work will establish a benchmark.

Although our model can also be compared to classical machine learning solutions, it has been observed in Ma et al. (2019) that machine learning models underperform compared to deep learning models across all benchmark datasets.

In conclusion, we have compared our models, GLULA-HAR and GLUSA-HAR, to various existing methods, considering their different architectures, performance metrics, and complexities.

### 5.4. The analysis of experimental results

In Table 5, the “Num. of Params” column provides four values representing the model sizes for the PAMAP2, SKODA, OPPORTUNITY, and USC-HAD datasets, respectively. If only one value is specified, it indicates that the network size remains relatively consistent with marginal changes across different datasets. The performance of the models is measured using the 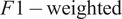 score, except for the Self-Att network (Mahmud et al., 2020), which utilizes the 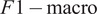 score. We also calculated the macro score for our proposed solution. However, for the DAPHNET dataset, we relied on the 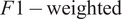 result presented in Tonmoy et al. (2021), given that both works are from the same author, ensuring the reliability of the score numbers. In the table, to account for the score type differences, two scores are presented in the format of 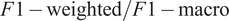 .

**Table 5.** Size and scores (F1-weighted/F1-macro) comparison of our model with listed methods on benchmark datasets. The bold values indicate the best or close to the best results for the corresponding metric in the table’s column among presented models.

	PAMAP2	SKODA	OPPORT.	USC-HAD	DAPH.	Num. of Params
GLULA	90.1/90.3	**97.6/96.3**	**95.9/78.0**	**59.4/50.9**	**94.1/79.7**	**50**/**51**/**196**/**4**/**4** K
GLUSA	89.9/88.0	**97.8/96.6**	95.5/72.2	54.3/46.5	92.4/72.8	*Same as above*
Lego–CNN (Tang et al., 2020)	91.4/	**–**	85.5/	**–**	**–**	2.86 M/**−**/610 k/**−**/**−**
Self–Att (Mahmud et al., 2020)	/**95.0**	/93.0	/61.0	/50.0	82.0/	590 K
DCL (Ordóñez and Roggen, 2016)	74.8/	91.2/	67.0/	38.0/	84.0/	331 K
Attsense (Ma et al., 2019)	89.3/	93.1/	**–**	**–**	**–**	331 K
HSA (Tonmoy et al., 2021)	**99.0**/	95.0/	68.0/	55.0/	85.0/	0.51/2.1/0.91/2.3/0.59 M
iSPLI (Ronald et al., 2021)	89.0/	**–**	88.0/	**–**	**94.0/**	1.34 M/**−**/1.35 M/**−**/1.33 M
R–CNN (Moya Rueda et al., 2018)	93.7/	**–**	92.1/	**–**	**–**	26 M/**−**/30 M/**−**/**−**

As shown, our proposed model, GLULA-HAR, achieves the highest scores in four benchmark datasets: USC-HAD, OPPORTUNITY, DAPHNET, and SKODA. GLUSA-HAR slightly outperforms the 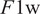 score for the SKODA dataset but falls behind in all other measurements.

On the PAMAP2 dataset, both models underperformed compared to R-CNN (Moya Rueda et al., 2018), HSA (Tonmoy et al., 2021), and Self-Att (Mahmud et al., 2020). The Self-Att network had 11 times more parameters than our solutions, while HSA had 10 times more parameters. GLULA-HAR had a slightly lower score than Lego-CNN but used 50 times fewer parameters than Lego-CNN (Tang et al., 2020). When comparing GLULA with Attsense (Ma et al., 2019), a linear-attention-based solution, GLULA had over six times fewer parameters and a higher 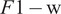 score by almost 1%.

Since the SKODA dataset is less complex than others, all presented algorithms achieved an F1 score of no less than 91%. Our model obtained the highest results among recent works while maintaining a substantially lower number of parameters. For example, HSA had 40 times more parameters, while Attsense had six times more.

For the OPPORTUNITY dataset, the difference in sizes was less significant, but the performance improvement was notable. There were evident contrasts in parameter count and performance between our solution and the classical DeepConvLSTM (Ordóñez and Roggen, 2016) as well as recent state-of-the-art models. Furthermore, Attsense, an expansion of DeepSense, which itself was based on DeepConvLSTM, exhibited a similar model size reduction compared to our proposed model.

In the USC-HAD dataset, our model outperformed the previous state-of-the-art models while containing significantly fewer parameters than the HSA and Self-Att networks, which showed similar performance. GLULA, with only 4,000 parameters, was much smaller compared to HSA, which had 2.3 million parameters.

The GLULA model for the DAPHNET dataset had an embedding dimension of 16, resulting in a model size almost equal to that of the USC-HAD case. The iSPLI network achieved an identical 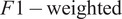 performance score of 94.0%. Our model performed slightly better by 0.1% while containing 300 times fewer parameters. The second closest model in terms of performance was HSA with an F1 score of 85%.

Overall, GLULA-HAR consistently achieves similar or higher scores than the softmax self-attention-based proposed model in all benchmark datasets. It also offers higher performance speed and lower complexity. Among all known models, GLULA-HAR achieves the highest 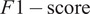 results in the USC-HAD, SKODA, DAPHNET, and OPPORTUNITY datasets, surpassing state-of-the-art models. Additionally, the proposed solution exhibits significantly fewer parameters than the presented networks.

### 5.5. The analysis of experimental results of LOSO cross-validation testing

To further demonstrate the validity of our proposed method, we conducted leave-one-subject-out cross-validation experiments on our model using each dataset, including SKODA. This approach involved excluding the data of one subject at a time for evaluation and repeating this process for each subject in the dataset. We then collected the results for each subject and calculated the average score across subjects. To account for randomness in the training, we performed the subject evaluation experiment five times with different seeds and took the mean score.

In the case of the SKODA dataset, which consists of only one subject, we divided the dataset into 10 random non-overlapping chunks to simulate the leave-one-subject-out cross-validation. We used the F1-weighted score as the evaluation metric and compared our proposed solution with a self-attention-based modification of the model, referred to as GLUSA. As shown in Table 6, GLULA outperforms GLUSA by a noticeable margin of about 1% in all datasets, except for USC-HAD and SKODA. However, when we consider the standard deviation of the performance across the evaluation trials, the difference between the proposed models in LOSO experiments is modest.

**Table 6.** LOSO-Cross-Validation F1-weighted scores comparison. The asterisk highlights the oversight in the reported result of the model’s experiment (row) on the corresponding dataset (column), where data leakage occurred in the form of labels being inadvertently included as part of the input.

	PAMAP2	SKODA	OPPORT.	USC-HAD	DAPH.
GLULA	74.8 ± 1.8	98.0 ± 0.5	79.0 ± 0.8	71.3 ± 4.3	86.2 ± 2.4
GLUSA	73.7 ± 2.1	98.0 ± 0.6	77.9 ± 0.4	72.4 ± 2.2	85.4 ± 1.9
HSA	94.0*	**–**	43.0	68.0	72.0
Self–Att	92.0*	**–**	42.0	60.0	71.0

To continue further on the performance evaluation of our proposed models in LOSO cross-validation testing, we incorporated results from other methods detailed in Section 5.3 for a comparative analysis with our models, namely GLULA-HAR and GLUSA-HAR. In particular, the models HSA (Tonmoy et al., 2021) and Self-Att (Mahmud et al., 2020) are presented in Table 6. This inclusion is because these models either outperform or are on par with other recent models (besides proposed methods) in terms of results on benchmark datasets, as shown in Table 5. A supplementary reason for adding these models is that their authors conducted LOSO cross-validation studies, in contrast to models like iSPLI (Ronald et al., 2021), which lack such results.

As evidenced by Table 6, both GLULA and GLUSA demonstrate superior performance during the LOSO cross-validation testing relative to HSA and Self-Att across three benchmark datasets: USC-HAD, OPPORTUNITY, and DAPHNET. Although the performance gap in the USC-HAD dataset is minimal, the LOSO results on the DAPHNET and OPPORTUNITY underline the noticeable advantage of GLULA over HSA and Self-Att in these datasets.

Similar to earlier analyses on benchmark test sets, Self-Att and HSA displayed commendable results on PAMAP2 LOSO cross-validation, with scores of 94.0 and 92.0%, respectively. Conversely, GLULA yielded a lower F1 of 74.8%. However, in the official implementation of HSA and Self-Att in the PAMAP2 dataset, a critical oversight was identified in the data preparation process. This misstep is present both in LOSO cross-validation experiments and benchmark set tests as referenced in 5.3. Specifically, this involved a form of data leakage where labels were inadvertently included as part of the input during inference. As mentioned earlier, this oversight should be addressed in future works.

Additionally, the performance of GLULA on the PAMAP2 LOSO cross-validation provided interesting insights into the dataset and the model’s adaptability to different subjects. Among the nine participants in the dataset, subjects 105 and 109, when used as test sets, exhibited the lowest performance scores. While no specific anomalies were identified in the data for subject 105, it is noteworthy that subject 109 was the only left-handed participant in the dataset (Reiss and Stricker, 2012). This particularity suggests that when the model was evaluated using subject 109 as the test set, it had been trained exclusively on data from right-handed participants, potentially contributing to the poor performance observed. This observation shows the need for future datasets to encompass a more diverse collection to better understand the effect of handedness on the model’s capacity to differentiate actions.

Regarding the SKODA dataset, as it was mentioned, there is only one subject present in the set. Consequently, it was not engaged in LOSO cross-validation testing for Self-Att and HSA. In contrast, we added outcomes from analogous experiments for SKODA to gather data for future comparative analyses.

It is important to note that the number of parameters remained consistent across these experiments, the same as in 5.4. This consistency reinforces the assertion that our proposed model boasts a significantly reduced parameter count compared to the recent works in the literature. This factor should be considered when evaluating models based on LOSO cross-validation.

A noteworthy observation from the results is the consistent drop in performance across all models when subjected to the LOSO cross-validation testing. This decline does not necessarily stem from flaws in the networks’ architecture or training. This phenomenon appears to be prevalent among the evaluated models. A plausible explanation for this observed trend could be attributed to uneven data distributions across individual subjects within the datasets. Such disparities might lead to irregular outcomes across differing participants. For instance, there exist scenarios where specific actions are absent for a given subject while being present in others for training. Such inconsistencies in action distribution across subjects can diminish the model’s performance on specific participants, culminating in a lowered aggregate score during LOSO cross-validation.

Considering the aforementioned observations and contrasting the outcomes of our proposed methodology with the latest state-of-the-art techniques, it becomes evident that GLULA possesses the comparative robustness to different subject-specific variability and the relative ability to adapt to some changes in the data distribution. Also, it is important to emphasize that benchmark test sets received more attention in this work because datasets’ authors tend to ensure a better balance between subjects and the representation of action classes in the test subset, thereby resulting in more reliable and robust evaluations.

### 5.6. Examination of inference speed

In Section 5.4, we highlighted that our proposed method, GLULA, has a notably reduced parameter count compared to recent models in the literature. Nonetheless, it is not immediately implied that it will also guarantee the fastest inference time. To further examine this point, we undertook experiments comparing the inference times between GLULA and recent state-of-the-art models, including HSA, iSPLI, and Self-Att. Our selection was driven by the fact that these networks showed the best or close to the best results in terms of performance scores, as evidenced in Table 5.

For our experiments, we used the official GitHub implementations of these models, modifying them specifically for inference purposes. We implemented changes to ensure the models operated solely in inference mode and further optimized both the models and their forward passes using Just-In-Time (JIT) compilation. This optimization was applied uniformly, including to our proposed model, to maintain consistent testing conditions. However, it is important to acknowledge that these conditions may not be an ideal environment for comparison, since there could be potential speed differences due to variability in computational graph implementations.

The inference time is significantly influenced by the window size (or duration) of the time-series input. An increase in the length of the time sequence notably elevates computational complexity, consequently slowing down the inference process. For instance, the complexity of a linear attention block increases linearly with the sequence length, while softmax self-attention, as previously discussed, scales quadratically. Table 7 illustrates variations in conditions relating to the configuration of the models’ input shapes. All dimensions presented in this table are determined post-completion of all of the pre-processing steps, just prior to model inference.

**Table 7.** Configuration of the models’ input dimensions following all pre-processing procedures across datasets

	PAMAP2	SKODA	OPPORT.	USC-HAD	DAPH.
GLULA	500, 40	250, 60	150, 113	100, 6	320, 9
Self–Att (Mahmud et al., 2020)	33, 18	49, 60	32, 77	32, 6	**–**
HSA (Tonmoy et al., 2021)	1500, 18	715, 60	300, 77	200, 6	320, 9
iSPLI (Ronald et al., 2021)	256, 36	**–**	90, 77	**–**	192, 9

*Note.* The first value in the table cell corresponds to the temporal dimension, and the second value represents the number of sensors’ channels.

The first value in each table cell denotes the length of the input sequence for a specific dataset and a certain model. Differences in sequence length primarily stem from the diverse pre-processing approaches employed in each study. For example, the Self-Att model employs a three-fold data downsampling, resulting in the shortest sequence length among the models compared. In contrast, in the other two works and our own, we directly utilize the datasets’ time steps. It is noteworthy that the Self-Att study (Mahmud et al., 2020) initially experimented with different window sizes similar in size to those in our work, but observed inferior performance. Consequently, all results used for the comparison are based on the dimensions that are listed in Table 7, which showed the best scores in their work.

The second value in the table denotes the total number of sensor channels utilized. In our proposed model, we employed nearly all channels available in the PAMAP2 and OPPORTUNITY datasets. This approach aimed to minimize hand-crafted preprocessing steps, allowing us to evaluate the model’s performance under those scenarios. On the other hand, in the Self-Att and HSA models, authors selectively used only certain channels, identified through empirical analysis as the most effective, while discarding others. It is important to note that the second dimension does not significantly impact computational complexity across datasets. This is because, in all the networks, the channel dimension is resized from the start using a learnable matrix (Mahmud et al., 2020; Tonmoy et al., 2021).

Additionally, not all datasets had corresponding implementations available, but despite this limitation, we could still recognize a general speed performance trend for the models on the available datasets. To facilitate a meaningful comparison between our leading model variant, GLULA, and the highlighted recent SOTA models, we conducted our evaluations on a device equipped with an NVIDIA RTX 3060 6GB GPU. This specific choice was made to have a constrained computational setting.

For the sake of precision and to ensure the results were not skewed by varying batch sizes, our experiments were designed to measure the inference time for individual data instances. To get an inference speed performance, we repeated this process 500 times for each model. Additionally, we initiated a GPU warm-up phase before starting our timed runs. Following all of that, we computed and recorded the average inference time for each model across the various datasets’ instance size parameters.

It is crucial to emphasize that the time measurements presented might exhibit variations when tested across diverse computing environments and hardware configurations. Additionally, while the speed of an optimized model can be influenced by the specifics of its implementation, our results underscore a recognizable trend. For clarity, all the time measurements in our study are expressed in milliseconds, where a lower value represents better performance.

Table 8 presents a comparative analysis of the average inference times of GLULA against existing techniques across benchmark datasets. As previously mentioned, we employed several optimization tweaks to enhance the inference speed of each model, and then JIT compiled them. These optimizations resulted in a noticeable reduction in inference times, which also brought the results closer numerically across different models. Additionally, it is important to note that all models compared in Table 8 are non-recurrent. This characteristic allows for parallelization across the temporal dimension, leading to smaller differences in inference times across varying input lengths, especially when using a batch size of one. For instance, GLULA demonstrated an average inference time of 0.94 ms on the PAMAP2 dataset, compared to 0.91 ms on the SKODA dataset, despite the sequence length of PAMAP2 being twice as long. It is also noteworthy that while the DAPHNET dataset has longer sequence lengths compared to the USC-HAD dataset, the processing time for DAPHNET was shorter. This is attributed to the fact that we doubled the dimensions in GLULA for the USC-HAD dataset, thereby affecting the inference time.

**Table 8.** The average inference time of the proposed model with existing methods on benchmark datasets for speed comparison

	PAMAP2	SKODA	OPPORT.	USC-HAD	DAPH.
GLULA	0.94 ± 0.05	0.91 ± 0.04	0.98 ± 0.04	0.9 ± 0.05	0.87 ± 0.03
Self–Att (Mahmud et al., 2020)	0.88 ± 0.07	0.89 ± 0.16	0.85 ± 0.07	0.86 ± 0.04	**–**
HSA (Tonmoy et al., 2021)	2.12 ± 0.23	8.32 ± 0.83	3.69 ± 0.47	1.42 ± 0.28	2.19 ± 0.38
iSPLI (Ronald et al., 2021)	1.36 ± 0.05	**–**	1.21 ± 0.06	**–**	1.41 ± 0.11

*Note.* The unit is ms.

In comparing the inference speed of GLULA with Self-Att, our proposed solution displayed only a marginal lag behind Self-Att across all datasets. For instance, in the SKODA dataset, Self-Att achieved an inference time of 0.89 ms, with GLULA trailing by 0.02 ms. This performance difference is attributable to the significant differences in input sequence lengths between the two models, as detailed in Table 7. In Self-Att., the input sequence length is considerably shorter than in GLULA – up to 15 times shorter in the case of PAMAP2 and 3 times shorter for USC-HAD. This variation arises due to the specific handcrafted preprocessing steps, such as downsampling, employed in Self-Att. It is important to note that the sequence length in Self-Att is fixed and does not vary like in GLULA, restricting the possibility of testing how varying lengths might impact inference speed. However, it can be assumed that with increased sequence lengths, Self-Att would either match or exceed the inference time of GLULA. This reason is based on the fact that while GLULA’s layers exhibit linear computational complexity relative to input length, Self-Att. incorporates two softmax self-attention sub-modules, each characterized by quadratic complexity (Mahmud et al., 2020).

GLULA consistently outperforms the other two models across all datasets in terms of inference speed. For instance, on the PAMAP2 dataset, GLULA registers a time of 0.94 ms, which is noticeably lower than HSA’s 2.12 ms., and iSPLI’s 1.36 ms. However, the main challenge for analysis is the varying input sizes for each of the datasets.

To establish a fair comparison between GLULA and HSA, we focused on their speed performance on the DAPHNET dataset, where the window sizes are identical, as shown in Table 7. In this context, GLULA noticeably outperforms HSA, with inference times of 0.87 and 2.19 ms, respectively. This substantial difference is primarily due to the complex hierarchy of softmax self-attention blocks integrated within HSA (Tonmoy et al., 2021). That implies multiple attention layers, each exhibiting quadratic complexity relative to the input length, which will be particularly sensitive when processing large window sizes without downsampling. Moreover, HSA shows varied time performance across different datasets due to its hierarchical structure being conditioned on the input sequence dimensions.

The input sequence lengths used in iSPLI’s for each dataset are approximately half of those in GLULA. Additionally, iSPLI is built on an inception convolutional network framework (Ronald et al., 2021), which has linear complexity in relation to the sequence input length. Despite these factors, GLULA consistently demonstrates faster inference speeds than iSPLI across all datasets. For example, in the OPPORTUNITY dataset, GLULA and iSPLI recorded inference times of 0.98 and 1.21 ms, respectively. This can be attributed to the inception-based network’s architecture, which involves numerous stacked layers of convolutions and consequently a larger number of parameters. That makes iSPLI overall more computationally intensive than GLULA, resulting in slower speed.

It is essential to remember that the efficiency of these models is tied to their implementation and the libraries used. Consequently, while the observed differences could be narrower or broader by better implementation, they underline GLULA’s potentially the fastest inference for individual data instances with respect to the input sequence length among recent SOTA works in the literature.

## 6. Conclusion

In conclusion, this article addressed the critical challenge of the size/performance trade-off in HAR for mobile and embedded systems, which is valuable in the production of monitoring systems that can follow human activity in real-time and control other systems based on that data. We introduced GLULA, a novel approach for HAR that combines linear attention, GCN, and wide convolutions while adapting structures such as branching. By using linear attention instead of regular softmax self-attention, our model achieved faster speed and reduced time and memory complexity. The incorporation of non-recurrence and parallelization further enhanced the flexibility and efficiency of our network.

To reduce the model’s parameter count, we avoided the use of feedforward layers and instead captured local dependencies more efficiently using GCN with branching. Additionally, by employing a prepended learnable token and a simple classification layer, we optimized parameter usage and minimized overhead. Our solution also employed various training techniques, including manifold mixup, one-cycle scheduling, and the AdaBelief optimizer, to enhance stability and handle limited data.

We conducted extensive experiments on five benchmark datasets (USC-HAD, PAMAP2, SKODA, DAPHNET, and OPPORTUNITY) to evaluate the performance, speed, and variations of our model. The original GLULA network, along with the suggested training techniques, demonstrated comparable or superior results compared to other variants. The linear attention-based method outperformed regular softmax-based approaches in HAR tasks while exhibiting lower time and space complexity.

Our experiments also revealed that the proposed network outperformed state-of-the-art models on the SKODA, USC-HAD, DAPHNET, and OPPORTUNITY datasets while maintaining the lowest parameter count by a noticeable margin and having one of the fastest inference time with regard to the input sequence length. This success can be attributed to the architectural structure of GLULA, which effectively captures local and global spatiotemporal features.

For future research, we suggest exploring the use of data generated by GANs and employing different augmentation techniques to further enhance the performance of HAR models. Additionally, considering the inspiration drawn from the Evolved Transformer and evolutionary-based neural architecture (AutoML) research, applying similar AutoML techniques to HAR tasks could lead to more robust solutions. Furthermore, the collection of a more diverse dataset would be advantageous in enhancing our understanding of the effects of handedness on action movements patterns and their differentiation.

## Data Availability

All datasets and sample codes used in this study were obtained from open-source resources and have universal access. The code for this study, along with the resulting weights and hyperparameters, will be made available at https://github.com/Etzelkut/HAR-proj. If required during the review process, the code and hyperparameters will be shared and/or added to the GitHub repository.
